# Tissue Damage in Human Cutaneous Leishmaniasis: Correlations Between Inflammatory Cells and Molecule Expression

**DOI:** 10.3389/fcimb.2020.00355

**Published:** 2020-07-14

**Authors:** Maíra Garcia Saldanha, Carla Pagliari, Adriano Queiroz, Paulo Roberto Lima Machado, Lucas Carvalho, Phillip Scott, Edgar M. Carvalho, Sérgio Arruda

**Affiliations:** ^1^Instituto Gonçalo Moniz, Fundação Oswaldo Cruz (FIOCRUZ), Salvador, Brazil; ^2^Departamento de Patologia, Faculdade de Medicina, Universidade São Paulo, São Paulo, Brazil; ^3^Serviço de Imunologia, Complexo Hospitalar Universitário Professor Edgard Santos, Universidade Federal da Bahia, Salvador, Brazil; ^4^Department of Pathobiology, School of Veterinary Medicine, University of Pennsylvania, Philadelphia, PA, United States; ^5^Departamento de Ciências de Vida, Universidade Estadual da Bahia, Salvador, Brazil

**Keywords:** cutaneous leishmaniasis, inflammatory cells, necrosis, amastigotes, tissue damage

## Abstract

Cutaneous leishmaniasis (CL) is caused by the bite of the infected sand fly, which inoculates parasites of *Leishmania* spp and triggers an immune response. An exacerbated cutaneous inflammatory response is crucial for controlling parasite burden but can also promote tissue damage. This study aimed to characterize the populations of natural killer (NK), CD57^+^, CD4^+^, and CD8^+^ T cells, CD20^+^ B cells, as well as CD68^+^ macrophages, in biopsies of ulcerated CL lesions, and quantify the production of perforin^+^, grazyme B^+^, interleukin 1 beta (IL-1β^+^) and Tumor Necrosis Factor (TNF-α^+^ cells). We then correlated these parameters with necrosis, inflammation and the number of amastigotes. CD4^+^ T cells were positively correlated to the extent of inflammation, B cells and IL-1β^+^ were associated with the extent of necrosis, CD68^+^ macrophages and perforin were correlated with the number of amastigotes, and CD57^+^ NK cells was correlated to CD68^+^ macrophages and amastigotes. In sum, the finding suggests that the production of cytotoxic granules and cytokines by inflammatory cells contributes to tissue damage in CL lesions.

## Introduction

Cutaneous leishmaniasis (CL) is the most common clinical form presented by individuals infected by protozoa of the genus *Leishmania*. After inoculation of *Leishmania Viannia braziliensis* in the skin by sandflies, a nodular lesion and an exuberant satellite lymphadenopathy is documented (Bomfim et al., 2007; Wind et al., 2014). The classical CL ulcers caused by *L. braziliensis* appear about 2–4 weeks after the presence of nodular lesions and are characterized by well-defined ulcer with raised borders. The evolution of CL is characterized by an exacerbated inflammatory response (Costa et al., 2018). In most infectious diseases, early treatment increases cure rates and decreases healing time; however, the introduction of therapy soon after infection in CL, i.e., prior to the appearance of ulcers, has been associated with a high rate of therapeutic failure (Machado et al., 2002; Unger et al., 2009; Khouri et al., 2014).

The main host defense mechanism against intracellular protozoa is the activation of macrophages by IFN-γ, mainly produced by CD4^+^ T cells (Santos et al., 2013). However as *Leishmania* are able to escape this killing mechanism, the persistence of the parasite and leishmanial antigens induce a marked inflammatory response that is associated with tissue damage and the development of skin ulcers (Santos et al., 2013). Several molecules have been associated with the pathology of *L. braziliensis* infection. Neutrophils are the cells that initially migrate after parasite inoculation, followed by macrophages (Novais et al., 2009; Conceição et al., 2016). The production of IFN-γ by NK cells may contribute to parasite killing, or may be cytotoxic, thereby contributing to this pathology (Muniz et al., 2016; Campos et al., 2017). Subsequently, the activation of CD4^+^ and CD8^+^ T cells is observed. T cell activation and the production of cytokines by these cells is determinant in the outcome of infection. An impairment in the host's Th1 immune response results in diffuse CL, which is characterized by multiple nodular lesions consisting predominantly of macrophages with a high parasite burden (Silveira et al., 2004). Alternatively, a normal Th1 immune response induces an exacerbated inflammatory response, leading to the presence of ulcerating lesions in CL (Bacellar et al., 2002; Antonelli et al., 2005; Castro Gomes et al., 2017).

IL-1β and TNF-α are highly expressed in CL ulcers, and may be involved in the tissue damage arising from *L. braziliensis* infection (Cardoso et al., 2015; Novais et al., 2017). The production of IL-1β by peripheral blood mononuclear cells is associated with ulcer size (Santos et al., 2013). Treatment with pentoxifylline, a drug that decreases TNF-α production, in combination with meglumine antimoniate, is more effective than antimony alone. Combined therapy not only reduces healing time in patients with mucosal leishmaniasis (ML), but also cures ML patients refractory to antimony therapy alone (Cuba et al., 1984; Lessa et al., 2001; Machado et al., 2002). Further data has indicated the participation of monocytes, CD4^+^ and CD8^+^ T cells, in the pathogenesis of CL, due to increased frequencies of intermediate monocytes referred to as an inflammatory monocyte subset. In CL and ML, macrophages present increased TLR expression, enhanced respiratory burst and produce higher levels of pro-inflammatory cytokines compared to cells from healthy subjects or individuals with subclinical *L. braziliensis* infection (Giudice et al., 2012; Carneiro et al., 2016; Muniz et al., 2016). With regard to the adaptive immune response, the size of CL ulcers is directly correlated with the frequencies of CD4^+^ T cells expressing IFN-γ and CD69, an early marker of T cell activation (Antonelli et al., 2005). More recently, the role of CD8^+^ T cells has been documented in the pathology of *L. braziliensis* in both mice and humans (Santos et al., 2013; Cardoso et al., 2015; Novais et al., 2018). Studies have shown that the killing of *L. braziliensis*-infected cells by CD8^+^ T cells leads to the increased production of IL-1β through the activation of the inflammasome, which may exacerbate tissue damage (Santos et al., 2013; Cardoso et al., 2015; Novais et al., 2018). However, most of these studies were conducted in mice or in human peripheral blood mononuclear cells, i.e., there is a paucity of data regarding the cells and molecules that participate in the pathogenesis of ulcer formation at the lesion site. Accordingly, the present study evaluated cell frequencies and quantified proinflammatory molecules in CL ulcer biopsies in an attempt to contribute to the knowledge surrounding the immunopathogenesis of CL lesions caused by *L. braziliensis*.

## Materials and Methods

### Patients

This study included 22 biopsies from 22 CL patients obtained punch biopsy samples (4 mm) after anesthesia from the border of ulcer and immediately fixed in 10% formalin-buffered solution. CL diagnosis was done by to the presence of amastigotes in these biopsies, as well as the detection of *L. braziliensis* DNA by PCR. Patient demographic characteristics, illness duration and lesion size were documented. All patients were subsequently treated with meglumine antimoniate (Glucantime®, Sanofi Aventis, Gentilly, France) at a dose of 20 mg/Kg.

### Immunohistochemistry

Deparaffinization and rehydration of 5-μm thick sections was performed using xylene and alcohol PA, followed by antigen retrieval with buffer pH 9.0 at 96°C for 20 min. Immunohistochemistry was conducted after blocking peroxidase activity with 3% hydrogen peroxide for 10 min, and protein activity with Protein Block Serum-Free (DAKO, Californie, USA) for 15 min. All slides were incubated at 4°C with the following antibodies: Monoclonal Mouse anti-CD57, clone Ab-1, Monoclonal Mouse anti-Perforin, clone 5B10 (ThermoFisher, Massachusetts, USA); Polyclonal Rabbit anti-TNF-α, clone orb129752 (Biorbyt, Cambridge, UK); Monoclonal Mouse anti-IL-1β, clone 3AC (Cell Signaling Technology, Massachusetts, USA); Polyclonal Rabbit anti-Granzyme B, clone EP230, Monoclonal Mouse anti-CD4, clone EP204, Monoclonal Mouse anti-CD8, clone C8/144B (Cell Marque, Californie, USA); Monoclonal Mouse anti-CD68, clone PG M1, Monoclonal Mouse anti-CD20, clone L26 (Dako, Californie, USA); Polyclonal Rabbit anti-*Leishmania* (in house) (Schubach et al., 2001). A Mouse and Rabbit Peroxidase Kit HorseRadish KP500 (Diagnostic BioSystems, Pleasanton, USA) was used to perform reactions according to manufacturer recommendations. All slides were counterstained with Harris hematoxylin, dehydrated and then mounted using Permount (Thermo Fisher Scientific, Massachusetts, USA) on glass coverslips.

### Quantitative Analysis

A Nikon 90i Eclipse microscope (Nikon Corporation, Tokyo, Japan) was coupled to a DS-Fi1 digital camera system (Nikon Corporation, Tokyo, Japan). All slides were photographed (five randomized fields from each section) at 40× and visualized with Nis-Elements software (v. 3.1). In each field, the number of positive cells was counted using the semiautomatic quantification feature of ImageJ software v. 1.48 (National Institutes of Health, Maryland, USA). Positive cells were identified via the amplified molecules that reacted with the chromogenic substrate DAB (eBioscience, Californie, USA). For all reactions, a preselected pattern was used as a positive control and sections that had not been incubated with the primary antibody were used as negative controls.

### Morphometry of Inflammation and Necrosis Areas

The histological sections stained with hematoxylin and eosin were scanned by an optical microscope (Olympus BX51, Tokyo, Japan). The total extension of these sections as well as the areas of inflammatory infiltrate and necrosis was measured by Image J 1.48v (National Institutes of Health). The total length of the biopsy fragment and the sum of the areas of inflammation and necrosis are shown in mm^2^. The percentage (%) of inflammation and necrosis in the biopsies were calculated by dividing total extension of inflammation and necrosis in mm^2^ by the total extension of the biopsy fragment multiplied by 100.

### Heatmap and Correlation Matrices

Language R through the RStudio interface (v1.2.5019) was used for plotting heatmaps and correlation matrices between the parameters and markers observed. The functions *corrplot* and *corrgram* was used to track the heatmaps and correlation matrices, respectively, using Pearson's statistical method. Positive correlations are displayed in blue and negative correlations in red color. Color intensity and the size of the circle are proportional to the correlation coefficients.

## Results

A total of 22 patients were enrolled with mean age of 40 years and 16 were male, corresponding to 73% of the study sample. Fifteen patients presented lymphadenopathy and lesions were more frequent in the lower limbs (86%) than the upper limbs (14%). The average duration of ulcers was 43 ± 25 days at the time of patient examination, with an average lesion size of 376 ± 336 mm^2^.

**Figure 1:**
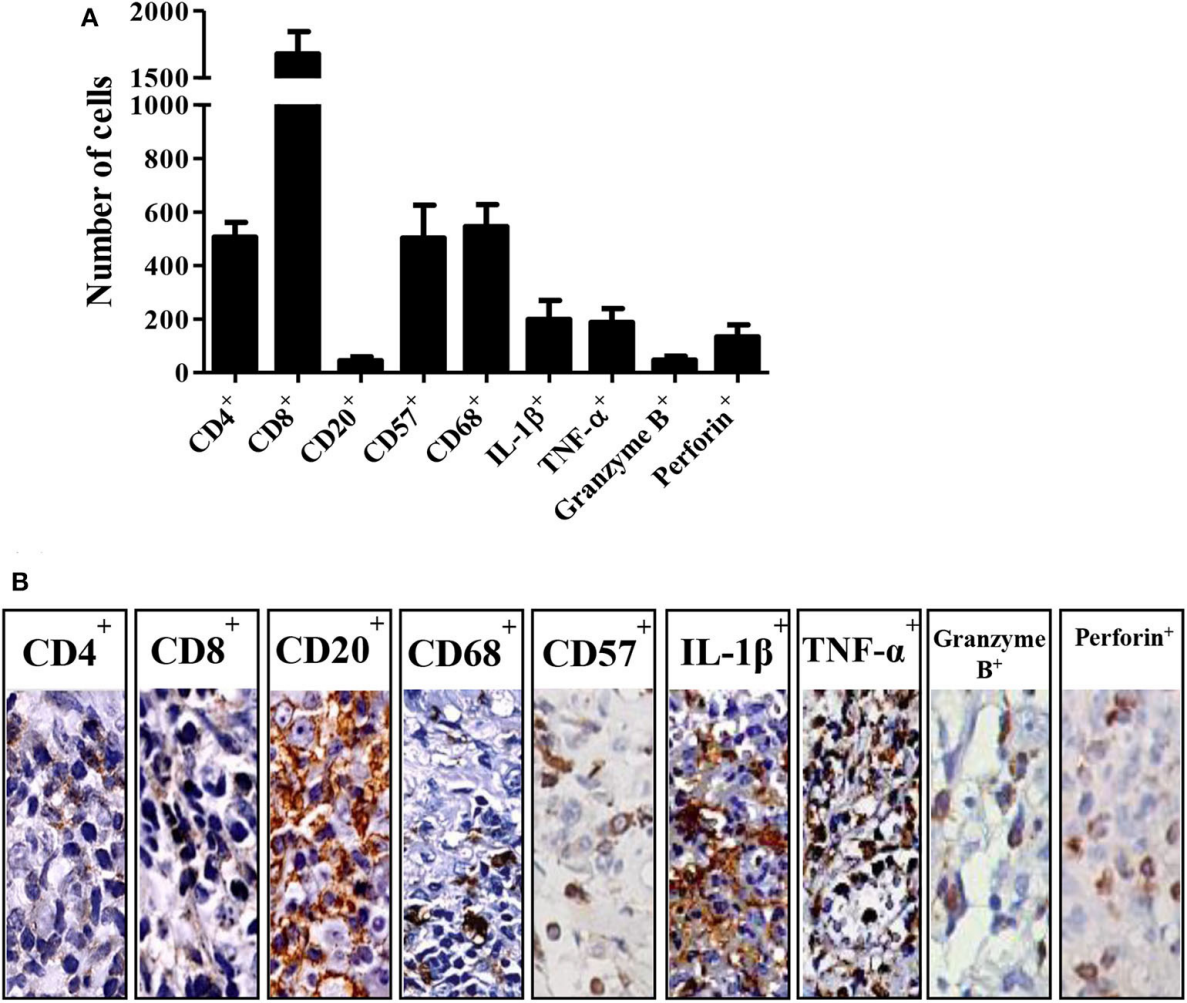
Cell profile in cutaneous leishmaniasis. **(A)** Number of positive cells by immunohistochemistry on biopsies from different patients with CL. The bars represent the standard mean error (SEM) (*n* = 22). **(B)** Representation of immunohistochemistry on CL biopsies.

Figure 1 illustrates the frequencies of cells expressing CD4^+^, CD8^+^, CD20^+^, CD57^+^, CD68^+^, IL-1β^+^, TNF-α^+^, granzyme B^+^ and perforin^+^ in tissue biopsies from CL patients. CD8^+^ cells were the most frequent, followed by CD68^+^, CD57^+^, and CD4^+^ (Figure 1A). Regarding the molecules secreted by these cells, IL-1β^+^ and TNF-α^+^ were found to be the most highly expressed, mainly in individuals with lymphadenopathy, followed by perforin^+^ and granzyme B^+^ (Figure 1B).

**Figure 2:**
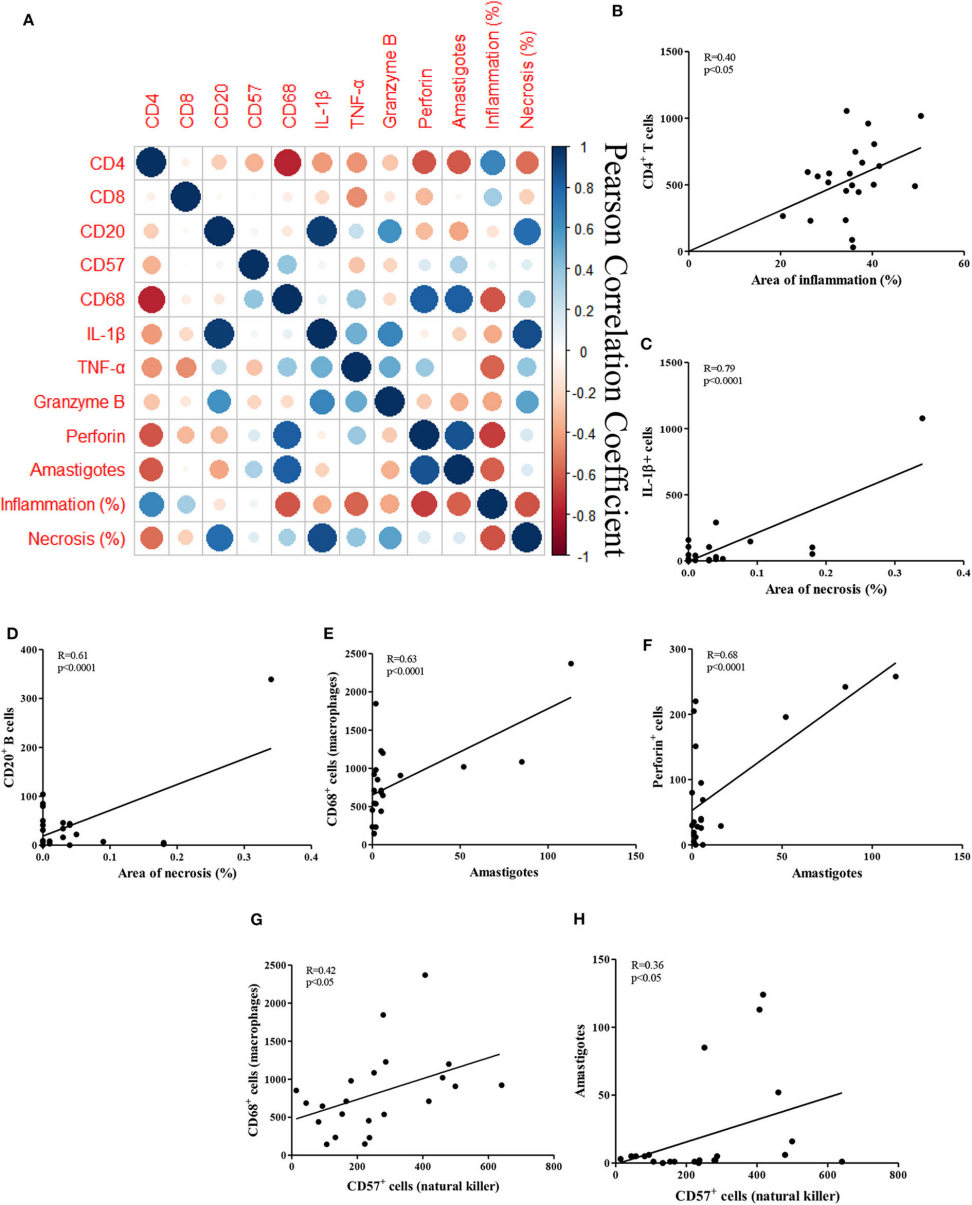
Correlations between histopathological parameters and cellular markers in CL biopsies. **(A)** Clustered heatmap of Pearson correlation coefficients of the inflammatory cells, inflammation and necrosis; **(B–H)** Pearson correlation of the cells, areas of inflammation and necrosis, amastigotes and NK cells; **(B)** CD4^+^ T cells vs. inflammation; **(C)** IL-1β^+^ cells vs. necrosis; **(D)** CD20^+^ B cells vs. necrosis; **(E)** CD68^+^ macrophages vs. amastigotes; **(F)** perforin^+^ cells vs. amastigotes **(G)** CD68^+^ macrophages vs. CD57^+^ NK cells; **(H)** amastigotes vs. CD57^+^ NK cells.

Figure 2 depicts correlations between the observed histopathological features and the frequencies of cells and molecules expressed by these cells, in addition to associated R values (Figure 2A). The inflammation seen in CL is characterized by the infiltration of mononuclear cells throughout the duration of the illness (Faria et al., 2009; Dantas et al., 2014). Increased infiltration is seen, ranging from mild in the early phase of disease, before the appearance of ulcers, which increases during ulcer development (Saldanha et al., 2017). Inflammation was found to be directly associated with the frequency of CD4^+^ T cells (*R* = 0.40; *P* = 0.05; Figure 2B).

Necrosis in CL ulcers are secondary to tissue damage due to the killing of macrophages infected with *L. braziliensis*, or the killing of cells expressing parasite antigens. However, the extent of necrosis in CL ulcers was reduced compared to the area of inflammation. No direct correlations were found between these two variables. However, IL-1β^+^ expression was strongly correlated with necrosis (*R* = 0.79; *P* < 0.0001; Figure 2C). Interestingly, the presence of B cells was also strongly correlated with necrosis (*R* = 0.61; *P* < 0.0001; Figure 2D).

Figures 2E,F shows the most frequent cells type and molecules associated with the presence of amastigotes in CL ulcers. As expected, the number of parasites was directly correlated with the presence of CD68^+^ macrophages (*R* = 0.63; *P* < 0.0001), as well as with the frequency of cells expressing perforin (*R* = 0.68; *P* < 0.0001).

Furthermore, there was no correlation of CD57^+^ NK cells with inflammation or necrosis, but these cells were positively correlated to CD68^+^ macrophages (*R* = 0.42; *P* < 0.05) and amastigotes numbers (*R* = 0.36; *P* < 0.05; Figures 2G,H).

## Discussion

The histological analysis of ulcerated lesions from patients with CL indicated chronic inflammation, with a predominance of mononuclear cellular infiltrate (Faria et al., 2009; Dantas, 2012). In the present study sample, CD4^+^ and CD8^+^ lymphocytes were highly expressed in ulcerative lesion biopsies. Both of these lymphocyte populations have been associated with protection and pathology. CD4^+^ T cells play a protective role through the production of IFN-γ, the main cytokine responsible for the activation of macrophages, which leads to intracellular parasite killing (Santos et al., 2013). Alternatively, CD8^+^ T cells have been more associated with tissue damage due to cytotoxic activity (Glennie and Scott, 2016; Castro Gomes et al., 2017). Here, we found cells expressing CD8^+^, CD4^+^ and CD68^+^ were the most frequent in tissue samples, and CD4^+^ cells were also positively correlated with the extent of inflammation. A previous study in CL patients demonstrated a direct correlation between the frequency of peripheral blood CD4^+^ T cells expressing IFN-γ, as well as TNF-α, and lesion size (Antonelli et al., 2005). It is well known that the production of IFN-γ by CD4^+^ T cells activates monocytes/macrophages and enhances the production of pro-inflammatory cytokines, which may contribute to the inflammatory response (Bacellar et al., 2002; Faria et al., 2009; Santos et al., 2013; Cardoso et al., 2015). Nonetheless, we found a negative correlation between CD4^+^ cells and CD68^+^ macrophages, CD4^+^ cells and the number of amastigotes and CD4^+^ cells and necrosis that are against a significant role of these cells in the pathology of CL.

CD8^+^ T cells were the most frequent cell type found in tissue biopsies from CL patients. Evidence indicates that these cells play an important role in the pathogenesis of *L. braziliensis* infection (Dantas et al., 2013; Novais et al., 2013). However, despite the high frequency of CD8^+^ T cells in the present tissue samples, we found no correlations between this cell population and the presence of amastigotes, nor inflammation or necrosis. This apparent discrepancy may be due to several factors. In fact, the production of granzyme and perforin molecules is more important than the frequency of CD8^+^ T cells, since these are directly involved in the lysing of target. In the tissue, granzyme was positively associated with necrosis and slightly negatively associated with amastigotes and inflammation, while perforin was strongly positively associated with amastigotes and slightly with necrosis, yet slightly negatively associated with inflammation. A large percentage of cells were found to express CD57^+^, a marker associated with highly cytotoxic CD8^+^ or NK cells (Kared et al., 2016). Moreover, CD57^+^ cells were the only cell type whose frequency was directly correlated with macrophages and amastigotes, suggesting the participation of CD57^+^ cells in killing infected macrophages.

Since *Leishmania* is an intracellular parasite, little emphasis has been placed on the presence of B cells and antibodies in the pathogenesis of the disease. However, B cells and plasma cells are documented in the tissue of CL patients, and anti-leishmanial antibodies are highly produced in severe of *Leishmania* spp. infection, such as the visceral form and diffuse cutaneous lesions (Bomfim et al., 2007; Rodriguez-Pinto et al., 2014). Nonetheless, the role of B cells and antibodies in the pathology of CL has not been well-studied. Opsonization by antibodies may enhance parasite uptake by macrophages. In addition, form immune complex deposition can contribute to tissue pathology. Here we found a strong direct correlation between CD20^+^ and IL-1β^+^ expression, as well as between CD20^+^ and the extent of necrosis, which highlights the need for future study to evaluate the role of B cells and antibodies in the pathogenesis of CL.

Necrotic cell death may contribute to granuloma formation, inflammation and tissue damage. The role of IL-1β^+^ and TNF-α^+^ has been well studied in in CL mouse models, and high levels of both cytokines are produced by peripheral blood mononuclear cells in CL patients (Antonelli et al., 2005; Campos et al., 2014; Passos et al., 2015). IL-1β^+^ can be produced by a variety of cells, including inflammatory monocytes, B lymphocytes, and epithelial and NK cells (Carvalho et al., 2012; Novais et al., 2017). Recently, IL-1β^+^, through inflammasome activation, as well as cytotoxic CD8^+^ T cells have both been associated with tissue damage in *L. braziliensis* infection (Novais et al., 2017). Here IL-1β^+^ and TNF-α^+^ were both more frequent in patients with lymphadenopathy, and were positively correlated with granzyme expression, and necrosis was also strongly directly correlated with IL-1β^+^, which suggests that this cytokines plays a key role in the pathology of CL.

Gene expression analysis in biopsied human CL ulcerative lesions revealed high expression of IL-1β^+^, TNF-α^+^, granzyme and perforin, as well as other molecules (Novais et al., 2017). The perforin and granzymes produced by NK cells and T lymphocytes promote the apoptosis of infected cells (Trapani and Smyth, 2002). Perforin forms pores in target cells and also participates in the upregulation of CD8 (Zhou, 2010), while granzymes lyse intracellular pathogens (Arias et al., 2014). Our *in situ* findings in human CL lesions suggest that the associations between inflammatory cells and the intense production of cytokines and cytotoxic products may contribute to necrosis and potentially participate in the formation of CL ulcers.

## Conclusion

This study attempted to establish correlations between histopathological CL parameters in order to elucidate the pathogenesis of this disease. We suggest that, in an effort to control parasites, infected macrophages are likely to remain in an activated state due to the production of proinflammatory cytokines by CD4^+^ T cells, in particular IL-1β^+^, thusly potentially contributing to tissue necrosis. In addition, the exacerbated production of cytotoxic granules containing perforin by CD8^+^ T cells and NK cells can aggravate tissue destruction, thereby intensifying skin ulcer formation.

## Data Availability Statement

All datasets generated for this study are included in the article/supplementary material.

## Ethics Statement

The studies involving human participants were reviewed and approved by Institutional Review Board (Instituto Gonçalo Moniz—IGM-FIOCRUZ, no. 533.032/2014). The patients/participants provided their written informed consent to participate in this study.

## Author Contributions

MS, SA, EC, and CP planned research and coordinated the study. MS, CP, and LC performed the experiments. PM and AQ provided the clinical samples. MS, EC, PS, and SA analyzed the data. MS and SA wrote the manuscript with contributions from EC, PS, and CP. All authors read and approved the submitted version of the manuscript.

## Conflict of Interest

The authors declare that the research was conducted in the absence of any commercial or financial relationships that could be construed as a potential conflict of interest.
